# Bacterial causes of otitis media with spontaneous perforation of the tympanic membrane in the era of 13 valent pneumococcal conjugate vaccine

**DOI:** 10.1371/journal.pone.0211712

**Published:** 2019-02-01

**Authors:** Corinne Levy, Emmanuelle Varon, Naim Ouldali, Alain Wollner, Franck Thollot, François Corrard, Andreas Werner, Stéphane Béchet, Stéphane Bonacorsi, Robert Cohen

**Affiliations:** 1 ACTIV, Association Clinique et Thérapeutique Infantile du Val-de-Marne, Saint Maur-des-Fossés, France; 2 GPIP, Groupe de Pathologie Infectieuse Pédiatrique, Paris, France; 3 AFPA, Association Française de Pédiatrie Ambulatoire, Saint-Germain-en-Laye, France; 4 Université Paris Est, IMRB-GRC GEMINI, Créteil, France; 5 Clinical Research Center (CRC), Centre Hospitalier Intercommunal de Créteil, Créteil, France; 6 National Reference Center for Pneumococci, Centre Hospitalier Intercommunal de Créteil, Créteil, France; 7 Unité d’épidémiologie clinique, Assistance Publique-Hôpitaux de Paris, Hôpital Robert Debré, ECEVE INSERM UMR 1123, Paris, France; 8 Université Paris Diderot, Sorbonne Paris Cité, Paris, France; 9 Service de Microbiologie, Assistance Publique-Hôpitaux de Paris, Hôpital Robert-Debré, 75019 Paris, France; 10 Unité Court Séjour, Petits nourrissons, Service de Néonatalogie, Centre Hospitalier Intercommunal de Créteil, Créteil, France; Instituto Butantan, BRAZIL

## Abstract

After pneumococcal conjugate vaccine (PCV) implementation, the number of acute otitis media (AOM) episodes has decreased, but AOM still remains among the most common diagnoses in childhood. From 2% to 17% of cases of AOM feature spontaneous perforation of the tympanic membrane (SPTM). The aim of this study was to describe the bacteriological causes of SPTM 5 to 8 years years after PCV13 implementation, in 2010. From 2015 to 2018, children with SPTM were prospectively enrolled by 41 pediatricians. Middle ear fluid was obtained by sampling spontaneous discharge. Among the 470 children with SPTM (median age 20.8 months), no otopathogen was isolated for 251 (53.4% [95% CI 48.8%;58.0%]): 47.1% of infants and toddlers, 68.3% older children (p<0.001). Among children with isolated bacterial otopathogens (n = 219), non-typable *Haemophilus influenzae* (NTHi) was the most frequent otopathogen isolated (n = 106, 48.4% [95% CI 41.6%;55.2%]), followed by *Streptoccocus pyogenes* (group A *streptococcus* [GAS]) (n = 76, 34.7% [95% CI 28.4%;41.4%]) and *Streptococcus pneumoniae* (Sp) (n = 61, 27.9% [95% Ci 22.0%;34.3%]). NTHi was frequently isolated in infants and toddlers (53.1%), whereas the main otopathogen in older children was GAS (52.3%). In cases of co-infection with at least two otopathogens (16.9%, n = 37/219), NTHi was frequently involved (78.4%, n = 29/37). When Sp was isolated, PCV13 serotypes accounted for 32.1% of cases, with serotype 3 the main serotype (16.1%). Among Sp strains, 29.5% were penicillin-intermediate and among NTHi strains, 16.0% were β-lactamase–producers. More than 5 years after PCV13 implementation, the leading bacterial species recovered from AOM with SPTM was NTHi for infants and toddlers and GAS for older children. In both age groups, Sp was the third most frequent pathogen and vaccine serotypes still played an important role. No resistant Sp strains were isolated, and the frequency of β-lactamase–producing NTHi did not exceed 16%.

## Introduction

Acute otitis media (AOM) remains a major public health problem in childhood worldwide; it is the leading bacterial infection in children and the first cause of antibiotics prescription in children in many countries [1, 2]. After pneumococcal conjugate vaccine (PCV) implementation, the number of pneumococcal and non-pneumococcal AOM episodes decreased [3, 4]. However, AOM still remains among the most common diagnoses in childhood [4].

To determine the bacteriological causes of AOM, the detection of bacterial species in middle ear fluid (MEF) obtained by tympanocentesis is the gold standard method [5, 6]. However, in many countries, mainly because the procedure is painful, tympanocentesis is not recommended or performed routinely for first-line AOM [7]. Indeed, to approach the bacterial etiology of AOM, two types of samples could be easily obtained: nasopharyngeal (NP) samples and MEF through an existing perforation [7–10]. NP carriage studies have several well-known limitations, but they have epidemiological value for monitoring serotypes and antibiotic resistance changes after PCV implementation [10, 11].

MEF samples obtained through an existing perforation could be contaminated by the external auditory canal flora during MEF collection [12, 13]. Indeed, the causative role of *Staphylococcus aureus*, *Pseudomonas aeruginosa* or *Turicella otitidis* in AOM is doubtful because these are normal inhabitants of the external auditory canal [13]. Therefore, only otopathogens such as *Streptococcus pneumoniae* (Sp), non-typable *Haemophilus influenzae* (NTHi), *Moraxella catharalis* and group A *Streptococcus* (GAS) must be considered if they grow in otorrhea samples [7, 9, 13, 14].

A recent study performed in several European countries showed that among AOM cases, approximately 7% featured spontaneous perforation of the tympanic membrane (SPTM) [15]. However, there were significant differences among countries, ranging from 2% of episodes in Italy and the United Kingdom to 17.2% in Sweden. These differences may be explained by differences in healthcare systems, guidelines and diagnostic criteria of AOM [16]. Before PCV13 implementation, since the beginning of this century, few studies in high-income countries have assessed the pathogens involved in otitis media with SPTM. We excluded studies from low-income countries because the frequency of otorrhea among otitis media and the clinical features substantially differed [17]. Brook et al. reported a cohort of 100 patients with otorrhea from 1993 to 2006 in the United States: the main pathogen was Sp, accounting for 49% of cases, followed by NTHi, 21%, and GAS, 11%. [18]. In Israel, before PCV7 implementation, Leibovitz et al. described a cohort of 822 children less than 3 years old who had AOM with otorrhea from 1996 to 2006: NTHi and Sp were the most frequent pathogens isolated (about 30% of bacteriologically proven cases), followed by GAS (less than 6% of cases) [19]. Stamboulidis et al. reported a large cohort of 5580 patients (<14 years old) with otorrhea in Greece from 2000 to 2008 (before and after PCV7 implementation) [20]: before PCV7 implemention, the first pathogen isolated was Sp (25%), followed by NTHi (20%) and GAS (12%). After PCV7 implementation, the main otopathogen was NTHi (16%), followed by Sp (13%) and GAS (12%). In Germany, van der Linden et al. reported data from 963 MEF obtained from children 2 months to 5 years of age with SPTM following PCV7 implementation [21]. They found that GAS was the main otopathogen isolated (11.7%) (more often in children ≥24 months) following by Sp (9.1%) and NTHi (6.5%) [21]. Finally, Marchisio et al. in Italy described a cohort of 458 children (median age 28 months) enrolled from 2001 to 2011 (before and after PCV7 implementation) [22]: NTHi was the most frequent pathogen isolated (51% of bacteriologically proven cases), followed by Sp (19%) and GAS (17%). Overall, Sp was the leading pathogen involved in otitis media with otorrhea before PCV implementation, GAS was more frequently involved in older children and NTHi was more frequent in younger children and in polymicrobial otorrhea [18–22]. However, PCVs also resulted in shifts to non-vaccine pneumococcal serotypes isolated from carriage or pneumococcal infections (invasive and non-invasive) [23].

In France, PCV13 was implemented in 2010, with high vaccination coverage (>92%) in the targeted population [24]. The aim of this study was to describe bacteriological causes of AOM with SPTM, 5 to 8 years after PCV13 implementation.

## Materials and methods

From October 2015 to January 2018, children aged 3 months to 15 years old with SPTM were prospectively enrolled by 41 pediatricians who are part of a research and teaching network (ACTIV, Association Clinique et Thérapeutique Infantile du Val de Marne [Clinical and Therapeutic Association of Val de Marne]) throughout France. For some patients, otorrhea was the first manifestation of AOM; for others, otorrhea occurred after AOM treatment failure or recurrence. Failure (non-responsive AOM) was defined as otorrhea appearing despite at least 48 hr of antibiotics or recurring less than 4 days after the end of antibiotic treatment. Recurrence was defined by the appearance of otorrhea 4 to 30 days after the end of antibiotic treatment for AOM [7].

After written informed consent was obtained, we queried parents or guardians regarding the child’s demographics, clinical symptoms and PCV immunization history. MEF was obtained by sampling spontaneous discharge according to clinical practice guidelines. MEF specimens were obtained with cotton-tipped wire swabs, immediately placed in transport medium (Copan Venturi Transystem, Brescia, Italy), and transported within 48 hr to one of the two centralized microbiology laboratories (Robert Debré Hospital or National Centre for Pneumococci at European Georges Pompidou Hospital, Paris, France). The study was approved by the Saint Germain en Laye Hospital Ethics Committee.

### Microbiology

MEF was observed by Gram staining and inoculated for bacterial growth. All plates (Columbia blood agar with and without Colistin-Nalidixic acid, and Polyvitex agar) were incubated with 5% CO_2_. A selective culture medium with Colistin-Nalidixic acid combination added was used for these non-sterile specimens because it allows for growth of Gram-positive bacteria, even in low quantity, in the presence of Gram-negative rods (particularly with a high inoculum). Bacterial isolates were identified by standard methods as described [7]. Sp serotyping and antibiotic susceptibility testing were performed at the National Reference Center for Pneumococci using the capsular swelling method with commercial antisera (Statens Serum Institut, Copenhagen, Denmark). Susceptibility of Sp isolates to penicillin G was determined by minimal inhibitory concentration (MIC) by the agar-dilution method as described [7, 10]. Isolates were divided into penicillin-susceptible (MIC ≤0.06 mg/L), penicillin-intermediate (MIC >0.06–2.0 mg/L) and penicillin-resistant (MIC >2 mg/L) according European Committee on Antimicrobial Susceptibility Testing breakpoints (Table version 7.1 available at http://www.eucast.org/clinicalbreakpoints/). *H*. *influenzae* isolates underwent capsular serotyping by the slide agglutination method with specific antisera (Phadebact, Boule Diagnostic, Hudinge, Sweden). The production of β-lactamase was assessed by a chromogenic cephalosporin test (Nitrocefin; Cefinase; Biomerieux, Marcy l’Etoile, France). NTHi strains were further classified as ampicillin-susceptible (MIC ≤ 1 mg/L) or -resistant (MIC > 1 mg/L). β-lactamase–negative ampicillin resistance (BLNAR) was determined according to the Clinical and Laboratory Standards Institute break points [25]. Strains were considered BLNAR strains if the 2-μg ampicillin diffusion test (Becton Dickinson) gave a zone of inhibition <20 mm and if the cefalotin disk diffusion test gave a zone of inhibition <17 mm.

#### Statistical analysis

Data were double-entered using 4D software (v6.4), and analyses were performed with Stata/SE v13.1 (StataCorp, College Station, TX, USA). Quantitative variables were compared by Student *t* test and categorical variables by chi-square test or Fisher exact test. All tests were two sided and the level of significance was set at p<0.05. Because most cases of SPTM due to GAS occurred in older children, we used the cut-off of 3 years to analyze our results [20, 22, 26, 27]. We used multivariate logistic regression analysis to identify factors related to MEF results. The variables age (<3 and ≥3 years), initial otorrhea, conjunctivitis, fever and otalgia were introduced into the models, and odds ratios [ORs] and 95% confidence intervals [95% CIs] were calculated.

## Results and discussion

From October 2015 to January 2018, 470 children with SPTM were enrolled: mean age was 28.8 ± 23.2 months (median 20.8 months) (Table 1). SPTM in children older than 3 years occurred mainly as the first manifestation of AOM (80.6%, n = 112) with otalgia (89.2%, n = 124). By contrast, for younger children (infants and toddlers), recurrence (22.1%, n = 73) and conjunctivitis (12.7%, n = 42) were more frequent (p = 0.013 for each result).

**Table 1:** Demographic characteristics of children with spontaneous perforation of the tympanic membrane.

	Total n = 470	<3 years old n = 331 (70.4%)	≥3 years old n = 139 (29.6%)	p value
Age (months), mean±SD Median	28.8±23.2 20.8	16.5±7.7 15.1	58.1±20.7 53.0	
Sex (m)	240 (51.1)	164 (49.6)	76 (54.7)	0.3
Day care center/school	349 (74.3)	212 (64.1)	137 (98.6)	
Child minder	60 (12.8)	60 (18.1)	0	
Home	61 (13.0)	59 (17.8)	2 (1.4)	
PCV13 vaccination	469 (99.8)	330 (99.7)	139 (100)	0.5
Antibiotic ≤ 3 days before MEF sample	9 (1.9)	8 (2.4)	1 (0.7)	0.2
Initial otorrhea	340 (72.3)	228 (68.9)	112 (80.6)	**0.01**
Non-responsive AOM	40 (8.5)	30 (9.1)	10 (7.2)	0.6
Recurrence of AOM	90 (19.2)	73 (22.1)	17 (12.2)	**0.013**
Temperature (°C), mean ±SD Median	37.9±0.9 37.6	37.9±0.9 37.6	37.9±0.9 37.6	0.6
No. of days since onset of otorrhea, mean ±SD Median	1.8±3.8 1	2.0±4.3 1	1.5±2.1 1	0.3
Otalgia	348 (74.0)	224 (67.7)	124 (89.2)	**<0.001**
Conjunctivitis	49 (10.4)	42 (12.7)	7 (5.0)	**0.013**

Data are n (%) unless indicated. PCV, pneumococcal conjugate vaccine; MEF, middle ear fluid; AOM, acute otitis media

### Overall population

For 251/470 cases, (53.4% [95% CI 48.8%;58.0%]), no otopathogen was cultured. Table 2 shows the microbiological results for MEF by age: 47.1% (n = 156/331) of infants and toddlers had a culture-negative result for the major bacterial pathogens, as did 68.3% (n = 95/139) of older children (p<0.001). In the overall population, NTHi was the most frequent otopathogen isolated (n = 106/219, 48.4% [95% CI 41.6%;55.2%]), followed by GAS (n = 76/219, 34.7% [95% CI 28.4%;41.4%]) and Sp (n = 61/219, 27.9% [95% CI 22.0%;34.3%]). NTHi was isolated more frequently in infants and toddlers (53.1%, n = 93/175), whereas for older children, the main otopathogen isolated was GAS (52.3%, n = 23/44). With co-infection with at least two otopathogens (16.9%, n = 37/219), NTHi was frequently involved (78.4%, n = 29/37).

**Table 2:** Microbiological results of middle ear fluid (MEF).

	Total n = 470	<3 years n = 331 (70.4%)	≥3 years n = 139 (29.6%)	p value
Absence of otopathogen	251 (53.4)	156 (47.1)	95 (68.3)	**<0.001**
At least ≥1 otopathogen	219 (46.6)	175 (52.9)	44 (31.7)	
Total NTHi	106 (48.4)	93 (53.1)	13 (29.5)	**0.005**
Total GAS	76 (34.7)	53 (30.3)	23 (52.3)	**0.006**
Total Sp	61 (27.9)	50 (28.6)	11 (25)	0.6
NTHi alone	77 (35.2)	69 (39.4)	8 (18.2)	**0.008**
GAS alone	63 (28.8)	41 (23.4)	22 (50)	**0.001**
Sp alone	37 (16.9)	30 (17.1)	7 (15.9)	0.8
Mc alone	5 (2.3)	4 (2.3)	1 (2.3)	1
Mixtures of otopathogens	37 (16.9)	31 (17.7)	6 (13.6)	0.4
NTHi+Sp	18 (8.2)	16 (9.1)	2 (4.6)	0.5
NTHi+ GAS	6 (2.7)	5 (2.9)	1 (2.3)	1
NTHi+Sp+Mc	3 (1.4)	2 (1.1)	1 (2.3)	0.5
NTHi+Mc	2 (0.9)	1 (0.6)	1 (2.3)	0.4
Mc+GAS	5 (2.3)	5 (2.3)	0	
Sp+GAS	2 (0.9)	2 (1.1)	0	
Sp+Mc	1 (0.5)	0	1 (2.3)	

Data are n (%). NTHi, non-typable *Haemophilus influenzae*; GAS, group A *streptococcus*; Sp, *Streptococcus pneumoniae*; Mc, *Moraxella catharalis*

Bacteriological results of MEF according to clinical characteristics showed that median age was significantly higher with GAS than NTHI and Sp (24.6 vs 18.3 and 17.1 months; p = 0.001). The association of SPTM with conjunctivitis was significantly more frequent with NTHi than Sp and GAS (18.2% vs 13.5% and 6.4%; p = 0.02). Otalgia was significantly less frequent with NTHi than Sp and GAS (61.0% vs 73.0% and 71.4%; p = 0.007).

On multivariate analysis, the main factors associated with NTHi isolated in MEF were conjunctivitis (adjusted OR [aOR] 2.0, 95% CI [1.0;4.0]), age less than 3 years (aOR 3.7, 95% CI [1.7;8.0]), and absence of otalgia (aOR 1.7, 95% CI [1.0;2.9]).

The serotype distribution (Table 3) showed that despite high vaccination coverage (>99.8%), PCV13 serotypes accounted for 32.1% (n = 18) of pneumococcal cases. Serotype 3 was the main serotype (16.1%, n = 9), followed, among the vaccine types (VTs), by 19F (8.9%, n = 5), 19A (5.4%, n = 3) and 18C (1.8%, n = 1). Non PCV13 serotypes (NVTs) were mostly represented by serotypes 23B (10.7%, n = 6), 11A (8.9%, n = 5), 15 B/C (7.2%, n = 4), 10A (5.4%, n = 3), 16F (5.4%, n = 3), 24F (5.4%, n = 3) and 35B (5.4%, n = 3). When Sp was isolated with other otopathogens, the frequency of NVTs was particularly high (86.4%, n = 18) as compared with Sp alone (55.9%, n = 19, p = 0.017).

**Table 3:** Distribution of serotypes by *Streptococcus pneumoniae* (Sp) infection alone or co-infection.

	Sp alone n = 34 (60.7%)	Sp with another otopathogen n = 22 (39.3%)
PCV13 serotypes (n = 18)	15 (44.1)*	3 (13.6)*
3 (n = 9)	8	1
19F (n = 5)	5	0
19A (n = 3)	2	1
18C (n = 1)	0	1
Non-PCV13 serotypes (n = 38)	19 (55.9)*	19 (86.4)*
23B (n = 6)	4	2
11A (n = 5)	2	3
15B/C (n = 4)	2	2
10A (n = 3)	0	3
16F (n = 3)	2	1
24F (n = 3)	2	1
35B (n = 3)	2	1
21 (n = 2)	1	1
23A (n = 2)	0	2
33F (n = 2)	1	1
31 (n = 2)	2	0
15A (n = 1)	1	0
8 (n = 1)	0	1
22F (n = 1)	0	1

Data are n (%). PCV, pneumococcal conjugate vaccine

*p = 0.017

Among Sp-infected cases, 29.5% strains were penicillin-intermediate isolates (no resistant strain); PCV13 serotypes accounted for 50% of these strains. Among NTHi strains, 16.0% were β-lactamase–producers and 7.5% ampicillin-resistant (BLNAR).

### Children with recurrent otorrhea

Recurrent otorrhea in the last 3 months accounted for 65 cases (13.8%), and most occurred in children less than 3 years old (n = 54, 83.1%) (Table 4). In this population, NTHi was the main otopathogen isolated (67.9%), followed by Sp (25.0%) and GAS (14.3%).

**Table 4:** Demographic characteristics of infants and toddlers and microbiological results of middle ear fluid (MEF) by recurrence of otorrhea in the last 3 months.

	Non-recurrent otorrhea n = 277 (83.7)	Recurrent otorrhea n = 54 (16.3)	P value
Age (months), mean ±SD Median	16.7±8.0 15.1	15.4±6.1 14.5	0.27
Sex (m)	135 (48.7)	29 (53.7)	0.5
Conjunctivitis	33 (11.9)	9 (16.7)	0.2
Otalgia	191 (69.0)	33 (61.1)	0.3
Temperature ≥38.5°C	99 (36.3)	15 (28.9)	0.3
Positive samples	147 (53.1)	28 (51.9)	0.9
Total NTHi	74 (50.3)	19 (67.9)	0.09
Total Sp	43 (29.3)	7 (25.0)	0.6
PCV13*	11 (28.2)	1 (14.3)	0.7
Non-PCV13*	28 (71.8)	6 (85.7)	
Total GAS	49 (33.3)	4 (14.3)	0.05
NTHi alone	53 (36.1)	16 (57.1)	**0.04**
GAS alone	37 (25.2)	4 (14.3)	0.2
Sp alone	26 (17.7)	4 (14.3)	0.8
Mixtures of otopathogens	28 (19.0)	3 (10.7)	0.4

* 4 missing data

NTHi, non-typable *Haemophilus influenzae*; GAS, group A *streptococcus*; Sp, *Streptococcus pneumoniae*; PCV, pneumococcal conjugate vaccine

This study describes the bacterial causes of otorrhea detected by culture methods performed more than 5 years after PCV13 implementation in a country with high PCV13 vaccination coverage. Before PCV13 implementation, the main pathogen isolated from acute spontaneous perforation was Sp, but our study shows that after PCV13 implementation, Sp was the third most common bacterial species isolated [18–20]. NTHi is the leading bacterium in infants and toddlers and GAS in older children [20, 28]. Moreover, we confirm that NTHi is frequently associated with other bacteria and also found frequently in young children with recurrent otorrhea (67.9%). This result strongly supports that pre-existing perforations are more likely associated with NTHi and/or polymicrobial culture, which suggests a role of biofilms [13, 28]. By contrast, GAS is preferentially detected in children ≥3 years old (52.3%) with otalgia, without preexisting perforation [16]. These findings are consistent with those previously published by Segal et al., who compared AOM caused by GAS and other otopathogens: GAS AOM was also characterized by a frequency of SPTM, older age of the child and, less frequently, recurrent AOM [21, 26].

Recently Pichichero et al. showed that PCV13 prevented AOM caused by Sp expressing VTs [29]. However, in our study, despite high vaccination coverage and PCV13 implementation lasting more than 5 years, some VTs (serotypes 3, 19F and 19A) were found in 32.1% of cases (all vaccinated), and NVTs accounted for 67.9%. Similar results were observed in Italy 4 years after PCV13 implementation, with 77.1% of NVTs [30]. Lewnard et al. recently found that PCV13 serotypes were among the most virulent in terms of progression from carriage to AOM, contrary to non-PCV13 serotypes, which explains in part the reduced benefit for incidence of AOM after PCV implementation, despite a complete serotype replacement in NP carriage [10, 31]. We found that with Sp isolated with other otopathogens, the frequency of NVTs was higher than with Sp alone (p = 0.017). These findings also support the results of Lewnard et al.: PCV13 serotypes were less frequently associated with NTHi than were non-PCV13 serotypes, which suggests that interactions with NTHi may change serotype-specific virulence for AOM [31]. Serotype 3 being the leading VT may indicate that this serotype is among the most virulent in terms of progression from carriage to AOM and for which a lower effectiveness of PCV13 has been demonstrated in other pneumococcal diseases [32, 33].

Our study has several limitations. The first was the high proportion of children without positive culture for any otopathogens (53.4%), but our data are consistent with previously published findings [34, 35]. The use of molecular diagnosis tools to detect bacteria and viruses in MEF could enhance the sensitivity as compared with culture performed in this study. Furthermore, co-infections with other pathogens could also be better detected with molecular methods [8, 12, 16, 28, 35]. The use of these techniques would have certainly increased both the proportion of proven causes of AOM, the rate of co-infection and viral infections. However, we were able to demonstrate the predominant role of NTHi in young children and, in cases of co-infection, the persistant role of PCV13 serotypes. Another limitation of our study was the small sample size for specific populations such as children with recurrent otorrhea (n = 54). Finally, the bacterial profile of AOM with otorrhea may not represent the broader spectrum of AOM: children with otorrhea may differ from those with an intact tympanic membrane, particularly for the major role of GAS in older children.

## Conclusions

In conclusion, 5 to 8 years after PCV13 implementation, NTHi is the leading bacterial species recovered from AOM with SPTM in infants and toddlers, and its role is increased with co-infection and recurrent AOM. In older children, GAS is the leading cause, and co-infections and reccurent AOM are rare. When Sp is implicated, VTs, particularly serotype 3, still play an important role. Antibiotic resistance of bacterial species involved in otorrhea was low: no resistant pneumococcal strains were isolated from MEF and the proportion of β-lactamase–producing NTHi did not exceed 16%. These results (bacterial profile and antibiotic resistance) could help with the antibiotic choice in AOM.
